# Supplementary material for: Acoustic enrichment can enhance fish community development on degraded coral reef habitat
Source: Nat Commun. 2019 Nov 29;10:5414. doi: 10.1038/s41467-019-13186-2 (PMC6884498; doi:10.1038/s41467-019-13186-2)
Supplement: Supplementary file 1 — Supplementary Information [file 41467_2019_13186_MOESM1_ESM.pdf]

Supplementary Information

For manuscript: Gordon *et al.,* Acoustic enrichment can enhance fish community development on degraded coral-reef habitat.

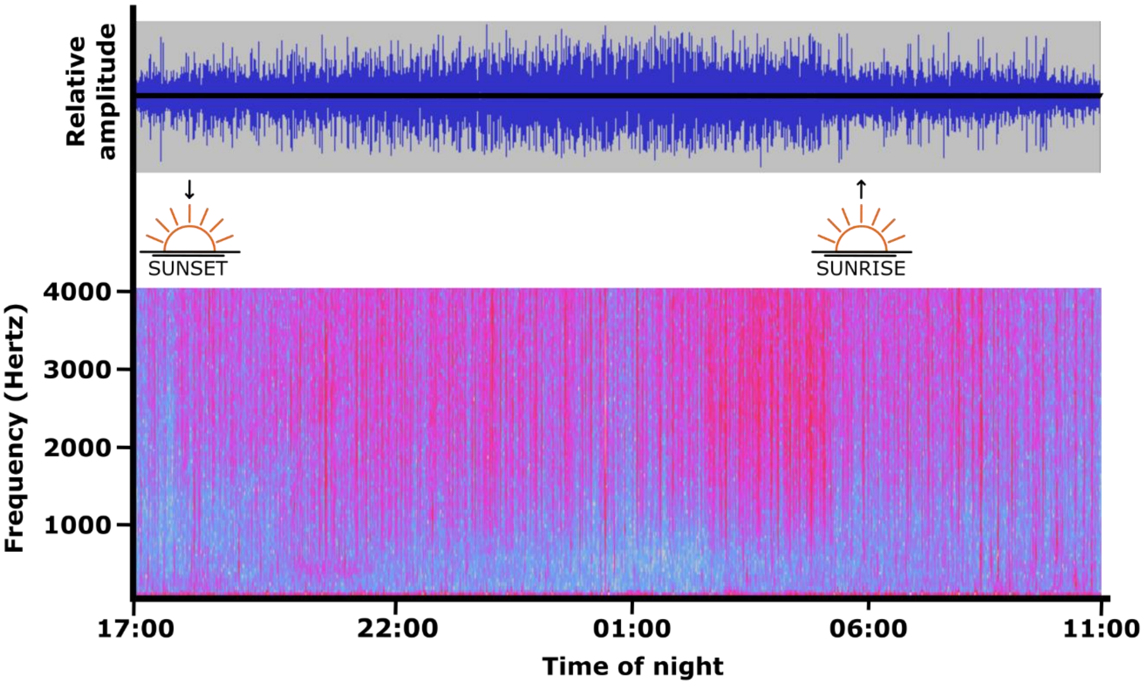

Supplementary Figure 1: A waveform (top) and spectrogram (bottom) of a representative example of the nocturnal reef noise playback. In the spectrogram, pink and red colours indicate a higher relative amplitude than blue colours; note the increase in acoustic energy after sunset and the decrease after sunrise.

Supplementary Table 1. Outputs from Linear Mixed Models (LMMs) and Generalised Linear Mixed Models (GLMMs) investigating abundance and diversity of fishes on experimental reefs after six weeks of loudspeaker playback of reef sound, dummy silent loudspeaker systems or no loudspeaker system. Effect sizes and standard error is provided for fixed effects; variance ± SD is provided for the random term 'track set'. Significant models and post-hoc comparisons ($p \leq 0.05$) are displayed in bold.

**Fixed effect (sound treatment)**	**Effect sizes ± SE**	**Post-hoc comparison**	**Tukey's HSD *p***
<u>**Pomacentridae abundance**</u> **(GLMM (Poisson family): χ^2 = 54.732, df = 2, p < 0.001)**			
Reef sound	18.518 ± 1.192	**Reef sound vs Dummy loudspeaker**	**< 0.001**
Dummy loudspeaker	9.003 ± 1.206	**Reef sound vs No loudspeaker**	**< 0.001**
No loudspeaker	9.684 ± 1.204	Dummy loudspeaker vs No loudspeaker	0.833
Track set random effect: 1.132 ± 1.423			
<u>**Herbivore abundance**</u> **(LMM: χ^2 = 7.499, df = 2, p = 0.024)**			
Reef sound	15.324 ± 1.975	**Reef sound vs Dummy loudspeaker**	**0.029**
Dummy loudspeaker	8.533 ± 1.975	**Reef sound vs No loudspeaker**	**0.049**
No loudspeaker	9.078 ± 1.975	Dummy loudspeaker vs No loudspeaker	0.977
Track set random effect: 1.467 ± 1.211			
<u>**Mixed diet abundance**</u> **(GLMM (poisson family): χ^2 = 53.53, df = 2, p < 0.001)**			
Reef sound	14.968 ± 1.210	**Reef sound vs Dummy loudspeaker**	**< 0.001**
Dummy loudspeaker	7.428 ± 1.227	**Reef sound vs No loudspeaker**	**< 0.001**
No loudspeaker	6.056 ± 1.235	Dummy loudspeaker vs No loudspeaker	0.384
Track set random effect: 1.157 ± 1.465			
<u>**Planktivore abundance**</u> **(GLMM (poisson family): χ^2 = 43.473, df = 2, p < 0.001)**			
Reef sound	10.222 ± 1.360	**Reef sound vs Dummy loudspeaker**	**< 0.001**
Dummy loudspeaker	4.702 ± 1.376	**Reef sound vs No loudspeaker**	**< 0.001**
No loudspeaker	4.946 ± 1.374	Dummy loudspeaker vs No loudspeaker	0.945
Track set random effect: 1.513 ± 1.904			

**Invertivore abundance (GLMM (poisson family): χ^2 = 31.857, df = 2, *p* < 0.001)**			
Reef sound	6.356 ± 1.156	**Reef sound vs Dummy loudspeaker**	**0.005**
Dummy loudspeaker	3.349 ± 1.201	**Reef sound vs No loudspeaker**	**< 0.001**
No loudspeaker	1.674 ± 1.281	**Dummy loudspeaker vs No loudspeaker**	**0.042**
Track set random effect: 1.021 ± 0.156			
**Piscivore abundance (GLMM (poisson family): χ^2 = 19.862, df = 2, *p* < 0.001)**			
Reef sound	2.686 ± 1.295	**Reef sound vs Dummy loudspeaker**	**0.006**
Dummy loudspeaker	0.961 ± 1.409	**Reef sound vs No loudspeaker**	**< 0.001**
No loudspeaker	0.641 ± 1.490	Dummy loudspeaker vs No loudspeaker	0.64
Track set random effect: 1.100 ± 1.361			
**Total abundance (LMM: χ^2 = 21.107, df = 2, *p* < 0.001)**			
Reef sound playback	54.983 ± 4.759	**Reef sound vs Dummy loudspeaker**	**< 0.001**
Dummy loudspeaker	27.490 ± 4.761	**Reef sound vs No loudspeaker**	**< 0.001**
No loudspeaker	24.763 ± 4.761	Dummy loudspeaker vs No loudspeaker	0.887
Track set random effect: 24.41 ± 4.94			
**Species richness (LMM: χ^2 = 12.848, df = 2, *p* = 0.002)**			
Reef sound	17.000 ± 0.987	**Reef sound vs Dummy loudspeaker**	**0.006**
Dummy loudspeaker	12.727 ± 0.987	**Reef sound vs No loudspeaker**	**< 0.001**
No loudspeaker	11.818 ± 0.987	Dummy loudspeaker vs No loudspeaker	0.792
Track set random effect: < 0.001 ± < 0.001			
**Effective Shannon diversity (LMM: χ^2 = 5.990, df = 2, *p* = 0.050)**			
Reef sound	12.197 ± 0.853	Reef sound vs Dummy loudspeaker	0.235
Dummy loudspeaker	10.335 ± 0.853	**Reef sound vs No loudspeaker**	**0.026**
No loudspeaker	9.225 ± 0.853	Dummy loudspeaker vs No loudspeaker	0.596
Track set random effect: 0.302 ± 0.549			

Supplementary Table 2. Trophic classification of reef fish, based on published literature[33,42–45] and FishBase[46]. When ID was completed only to family level (e.g. 'Unknown goby 1'), trophic classifications were assigned to all species from that family known to occur in the Lizard Island area (using the Lizard Island Field Guide, available at lifg.australianmuseum.net.au), and the most commonly occurring trophic classification in the family was chosen. Corallivores made up less than 0.25% of all fish, and were found on 2 of 33 reefs; they were therefore excluded from subsequent analysis due to a lack of statistical power. All other trophic guilds represented at least 4% of the total count, and were found on at least 19 of 33 reefs.

Diet	Family	Species
Corallivore	Chaetodontidae	*Chaetodon auriga*
Corallivore	Chaetodontidae	*Chaetodon melanotus*
Herbivore	Acanthuridae	*Acanthurus dussumieri*
Herbivore	Acanthuridae	*Acanthurus nigricauda*
Herbivore	Acanthuridae	*Acanthurus xanthopterus*
Herbivore	Acanthuridae	*Naso tonganus*
Herbivore	Blenniidae	*Salarias fasciatus*
Herbivore	Pomacentridae	*Dischistodus perspicillatus*
Herbivore	Pomacentridae	*Plectroglyphidodon leucozonus*
Herbivore	Pomacentridae	*Pomacentrus adelus*
Herbivore	Pomacentridae	*Pomacentrus bankanensis*
Herbivore	Pomacentridae	*Pomacentrus chrysurus*
Herbivore	Pomacentridae	*Pomacentrus pavo*
Herbivore	Pomacentridae	*Pomacentrus wardi*
Herbivore	Scaridae	*Chlorurus spilurus*
Herbivore	Scaridae	*Scarus frenatus*
Herbivore	Siganidae	*Siganus doliatus*
Herbivore	Siganidae	*Siganus spinus*
Invertivore	Balistidae	*Abalistes stellaris*
Invertivore	Balistidae	*Rhinecanthus aculeatus*
Invertivore	Balistidae	*Sufflamen chrysopterum*
Invertivore	Gobiidae	*Amblygobius phaleana*
Invertivore	Holocentridae	*Sargocentron diadema*
Invertivore	Labridae	*Cheilinus chlorourus*
Invertivore	Labridae	*Coris batuensis*
Invertivore	Labridae	*Cymolutes torquatus*
Invertivore	Labridae	*Halichoeres trimaculatus*
Invertivore	Labridae	*Labroides dimidiatus*
Invertivore	Labridae	*Stethojulis strigiventer*

Invertivore	Mullidae	*Parupeneus barberinus*
Invertivore	Mullidae	*Parupeneus multifasciatus*
Invertivore	Nemipteridae	*Scolopsis lineata*
Invertivore	Syngnathidae	*Corythoichthys intestinalis*
Invertivore	Syngnathidae	*Corythoichthys ocellatus*
Mixed diet	Apogonidae	*Apogon doederleini*
Mixed diet	Apogonidae	*Apogonichthyoides melas*
Mixed diet	Apogonidae	*Cheilodipterus quinquelineatus*
Mixed diet	Apogonidae	*Fowleria marmorata*
Mixed diet	Apogonidae	*Ostorhinchus neotes*
Mixed diet	Apogonidae	*Ostorhinchus nigrofasciatus*
Mixed diet	Apogonidae	*Ostorhinchus properuptus*
Mixed diet	Apogonidae	*Pristiapogon exostigma*
Mixed diet	Apogonidae	*Pristicon trimaculatus*
Mixed diet	Blenniidae	*Meiacanthus grammistes*
Mixed diet	Blenniidae	*Meiacanthus lineatus*
Mixed diet	Gobiidae	*Amblygobius phalaena*
Mixed diet	Gobiidae	Unknown goby 1
Mixed diet	Gobiidae	Unknown goby 2
Mixed diet	Gobiidae	*Valenciennea longipinnis*
Mixed diet	Gobiidae	*Valenciennea puellaris*
Mixed diet	Labridae	Unknown wrasse 1
Mixed diet	Pinguipedidae	*Parapercis australis*
Mixed diet	Pinguipedidae	*Parapercis lineopunctata*
Mixed diet	Pomacentridae	*Pomacentrus tripunctatus*
Mixed diet	Scorpionidae	*Dendrochirus brachypterus*
Mixed diet	Scorpionidae	*Dendrochirus zebra*
Mixed diet	Scorpionidae	*Parascorpaena armata*
Mixed diet	Serranidae	*Grammistes sexlineatus*
Mixed diet	Tetraodontidae	*Canthigaster bennetti*
Mixed diet	Tetraodontidae	*Canthigaster papua*
Mixed diet	Tetraodontidae	*Canthigaster valentini*
Piscivore	Lethrinidae	*Lethrinus nebulosus*
Piscivore	Lutjanidae	*Lutjanus gibbus*
Piscivore	Muraenidae	*Gymnothorax undulatus*
Piscivore	Pseudochromidae	*Pseudochromis fuscus*
Piscivore	Serranidae	*Epinephelus quoyanus*
Planktivore	Chaetodontidae	*Heniochus acuminatus*
Planktivore	Pomacentridae	*Chrysiptera flavipinnis*
Planktivore	Pomacentridae	*Dascyllus aruanus*
Planktivore	Pomacentridae	*Pomacentrus amboinensis*
Planktivore	Pomacentridae	*Pomacentrus coelestis*
Planktivore	Pomacentridae	*Pomacentrus nagasakiensis*
