# Supplementary material for: Acoustic enrichment can enhance fish community development on degraded coral reef habitat
Source: Nat Commun. 2019 Nov 29;10:5414. doi: 10.1038/s41467-019-13186-2 (PMC6884498; doi:10.1038/s41467-019-13186-2)
Supplement: Supplementary file 3 — Reporting Summary [file 41467_2019_13186_MOESM3_ESM.pdf]

nature research

Corresponding author(s): Timothy A. C. Gordon (tg333@exeter.ac.uk)

Last updated by author(s): Oct 10, 2019

Reporting Summary

Nature Research wishes to improve the reproducibility of the work that we publish. This form provides structure for consistency and transparency in reporting. For further information on Nature Research policies, see Authors & Referees and the Editorial Policy Checklist.

Statistics

For all statistical analyses, confirm that the following items are present in the figure legend, table legend, main text, or Methods section.

n/a	Confirmed	
☐	☒	The exact sample size (*n*) for each experimental group/condition, given as a discrete number and unit of measurement
☐	☒	A statement on whether measurements were taken from distinct samples or whether the same sample was measured repeatedly
☐	☒	The statistical test(s) used AND whether they are one- or two-sided<br>*Only common tests should be described solely by name; describe more complex techniques in the Methods section.*
☐	☒	A description of all covariates tested
☐	☒	A description of any assumptions or corrections, such as tests of normality and adjustment for multiple comparisons
☐	☒	A full description of the statistical parameters including central tendency (e.g. means) or other basic estimates (e.g. regression coefficient) AND variation (e.g. standard deviation) or associated estimates of uncertainty (e.g. confidence intervals)
☐	☒	For null hypothesis testing, the test statistic (e.g. *F*, *t*, *r*) with confidence intervals, effect sizes, degrees of freedom and *P* value noted<br>*Give P values as exact values whenever suitable.*
☒	☐	For Bayesian analysis, information on the choice of priors and Markov chain Monte Carlo settings
☒	☐	For hierarchical and complex designs, identification of the appropriate level for tests and full reporting of outcomes
☐	☒	Estimates of effect sizes (e.g. Cohen's *d*, Pearson's *r*), indicating how they were calculated

Our web collection on statistics for biologists contains articles on many of the points above.

Software and code

Policy information about availability of computer code

Data collection	No software was used for data collection in this study.
Data analysis	All figure creation and statistical modelling was conducted in R v. 3.5.0. Figures were prepared using the packages cowplot, ggmap and ggplot2. Statistical modelling was conducted using the packages lme4 and mgcv. Acoustic recordings were analysed using the paPAM and PAMGuide packages on MATLAB.

For manuscripts utilizing custom algorithms or software that are central to the research but not yet described in published literature, software must be made available to editors/reviewers. We strongly encourage code deposition in a community repository (e.g. GitHub). See the Nature Research guidelines for submitting code & software for further information.

Data

Policy information about availability of data

All manuscripts must include a data availability statement. This statement should provide the following information, where applicable:

- Accession codes, unique identifiers, or web links for publicly available datasets
- A list of figures that have associated raw data
- A description of any restrictions on data availability

Raw data are available from the University of Exeter’s institutional repository.

Field-specific reporting

Please select the one below that is the best fit for your research. If you are not sure, read the appropriate sections before making your selection.

☐ Life sciences ☐ Behavioural & social sciences ☒ Ecological, evolutionary & environmental sciences

For a reference copy of the document with all sections, see nature.com/documents/nr-reporting-summary-flat.pdf

Ecological, evolutionary & environmental sciences study design

All studies must disclose on these points even when the disclosure is negative.

Study description	Thirty-three experimental coral-rubble patch reefs were constructed on Australia's northern Great Barrier Reef. Each reef was assigned randomly to one of three treatments in an independent-measures design, within the constraints that there were equal numbers of reefs in each treatment and the same treatment was never allocated to reefs that were spatially adjacent. The three treatments were soundscape-restored reefs (healthy soundscapes broadcast from loudspeakers overnight), dummy-loudspeaker reefs (a silent loudspeaker replica attached to the reef in the same way as loudspeakers), and no-loudspeaker reefs. Developing fish communities on each reef were monitored for 40 days, with juvenile damselfishes surveyed regularly throughout the 40 days, and the entire juvenile fish community surveyed once at the end of the 40 days.
Research sample	Communities of juvenile damselfishes associated with 33 experimental coral-rubble patch reefs (n = 11 for each treatment) were sampled throughout the experimental period; damselfishes were chosen for this as they are non-cryptic, highly abundant (up to 50% of reef fish communities) and possible to visually survey accurately with minimal disturbance to the developing fish community. Full community surveys of all juvenile fishes associated with the same reefs were carried out at the end of the experimental period (33 reefs, n = 11 for each treatment).
Sampling strategy	Experimental coral-rubble reefs were built in sandy areas of 2–4.5 m depth, at a minimum of 100 m from each other. The minimum distance between reefs was first chosen based on measurements of loudspeaker playback acoustic propagation in both sound-pressure and particle-motion domains. Having determined this, the maximum possible number of experimental reefs in the space available at the field site was 33 (n = 11 per treatment); this was therefore the sample size.
Data collection	For surveys of damselfishes throughout the experiment, visual surveys by a SCUBA diver (T.A.C.G.) were carried out, with the observer and dive buddy maintaining a distance of at least 1 m from the reef during surveys in order to minimise disturbance to the community. For surveys of the whole community at the end of the experiment, each reef was surveyed by the observer (T.A.C.G.) dismantling the reef piece-by-piece, checking each piece of rubble thoroughly and using dilute clove oil and a hand net to capture all juvenile fishes on the reef. All fishes were identified to species, except in cases where uncertainty meant that identification was only possible to family or sub-family level. In both cases, data were recorded on a dive slate.
Timing and spatial scale	All reefs were constructed between 27 October and 5 November 2017. Forty-day survey periods were started for each reef immediately after construction, with final community surveys taking place between 6 December and 15 December. Regular surveys of damselfishes (family Pomacentridae) were carried out throughout the 40-day period; each reef was surveyed 10 times, with between 3–9 days between consecutive surveys. Each reef was a minimum of 100 m from its nearest neighbour, with all reefs placed on sand flats in water depth of 2–4.5 m in the lagoonal waters south and south-west of Lizard Island Research Station.
Data exclusions	No data were excluded from the final whole-community analyses or the regular damselfish surveys. In the trophic-level analyses, corallivores made up less than 0.25% of all fish, and were found on only two of 33 reefs; due to a lack of statistical power, they were therefore not analysed as a separate trophic group in the manner that other groups were (herbivores, piscivores, planktivores etc.).
Reproducibility	This experiment is the first to use this methodology for long-term community development surveys. However, short-term deployment of patch reefs using the same protocol is a well-established method in the literature, and has been experimentally replicated many times (e.g. Simpson et al. 2005, Science 305; Radford et al. 2011, Coral Reefs 30; Gordon et al., 2018, PNAS 115).
Randomization	Loudspeaker treatment was allocated randomly to experimental reefs, but constrained such that there were equal numbers of reefs in each treatment, and the same treatment was never allocated to reefs that were spatially adjacent. Within the soundscape-restoration treatment, five different playback tracks were used to reduce pseudoreplication, with each track being allocated to soundscape-restoration reefs at random (each recording was allocated to 2–3 of the 11 soundscape-restored reefs). Playback track ID was included as a random term in linear mixed model and generalised linear mixed model analyses.
Blinding	It was not possible to be blind to the experimental treatment during surveys, as field surveys were carried out in situ on reefs where the loudspeaker, dummy loudspeaker or absence of loudspeaker was evident to the surveyor.

Did the study involve field work? ☒ Yes ☐ No

Field work, collection and transport

Field conditions	Weather conditions were calm during the experiment; on 88% of the days, wind speeds were less than 15 knots, on the remaining 12% of days, wind speeds were between 15 and 30 knots. Sea state was always 0–2 on the Beaufort scale. As all different treatments were being run simultaneously, any variation in weather conditions would be experienced equally by all treatments.

Location	This study was carried out in the lagoon south-west of Lizard Island Research Station, Australia (14°40.8'S, 145°26.4'E). Lizard Island is a continental mid-shelf island in the northern Great Barrier Reef with an extensive surrounding fringing and lagoonal reef system. Experimental reefs were constructed in 2–4.5 m mid-tide depth; maximum tidal range during the experiment was ±1.3 m.
Access and import/export	Permission and ethical approval for this experiment were granted by Lizard Island Research Station, the Great Barrier Reef Marine Park Authority (G17/39752.1), James Cook University (A2408, A2361), and the University of Exeter (2013/247).
Disturbance	During the experimental period, disturbance to fish communities caused by surveyors was minimised by both the observer and dive buddy maintaining a distance of at least 1 m from reefs. After the experimental period, all fishes were captured and released unharmed onto nearby reefs. Rubble-patches were then dismantled and removed from sand flats, and rubble was returned to the location of collection.

Reporting for specific materials, systems and methods

We require information from authors about some types of materials, experimental systems and methods used in many studies. Here, indicate whether each material, system or method listed is relevant to your study. If you are not sure if a list item applies to your research, read the appropriate section before selecting a response.

Materials & experimental systems

n/a	Involved in the study
☒	☐ Antibodies
☒	☐ Eukaryotic cell lines
☒	☐ Palaeontology
☐	☒ Animals and other organisms
☒	☐ Human research participants
☒	☐ Clinical data

Methods

n/a	Involved in the study
☒	☐ ChIP-seq
☒	☐ Flow cytometry
☒	☐ MRI-based neuroimaging

Animals and other organisms

Policy information about studies involving animals; ARRIVE guidelines recommended for reporting animal research

Laboratory animals	This study did not involve laboratory animals.
Wild animals	Wild communities of juvenile fishes recruiting to experimental coral-rubble patch reefs were observed, photographed, counted and recorded for 40 days. This was done without disturbance (divers maintaining a distance of 1 m from reefs) for surveys within the 40-day experimental period. During final surveys at the end of the 40-day experimental period, fishes were captured using dilute clove oil and hand nets, and transported in hand nets underwater by SCUBA divers to nearby reefs, where they were released unharmed. Following this, coral-rubble patch reefs were dismantled, and rubble was returned to the location from which it was collected.
Field-collected samples	No samples were collected from the field during this study.
Ethics oversight	Permission and ethical approval for this experiment were granted by Lizard Island Research Station, the Great Barrier Reef Marine Park Authority (G17/39752.1), James Cook University (A2408, A2361), and the University of Exeter (2013/247).

Note that full information on the approval of the study protocol must also be provided in the manuscript.
